# Linguistic and cultural validation of symptom questionnaire for visual dysfunctions (SQVD) for psychometric analysis in the Chinese context

**DOI:** 10.1097/MD.0000000000039459

**Published:** 2024-08-30

**Authors:** Yufeng Wang, Zizhong Zhang, Weiwei Jiang, Hongai Liu, Xin Jia, Xianrong Yang

**Affiliations:** a Inner Mongolia Medical University, Hohhot, Inner Mongolia, China; b Graduate Office, Changzhi Medical College, Changzhi, China; c Jining Institute of Education, Jining, China; d Department of Pharmacy, The Affiliated Hospital of Inner Mongolia Medical University, Hohhot, Inner Mongolia, China; e Department of Pharmacy, The Affiliated Hospital of Inner Mongolia Medical University, Hohhot, Inner Mongolia, China; f Department of Ophthalmology, The Affiliated Hospital of Inner Mongolia Medical University, Hohhot, Inner Mongolia, China.

**Keywords:** questionnaires, Rasch analysis, SQVD, validation

## Abstract

The Spanish scale symptom questionnaire for visual dysfunctions (SQVD) was sinicized and tested for reliability and validity in the Chinese context, employing both classical measurement theory and item response theory. A meticulous translation was conducted using the modified Brislin translation model, with input from experts for cross-cultural debugging and in-depth review. Following a pre-survey study, the Chinese version of the SQVD was finalized. A convenience sampling method was used to select 270 patients from the target group and 252 valid questionnaires were successfully collected. The Rasch model was employed to assess response category functionality, fit statistics, unidimensionality, person and item reliability, separation, targeting, and differential item functioning. Classical test theory was applied to evaluate internal consistency and retest reliability, supplemented by correlation analysis. Job characteristic curves were also plotted to assess diagnostic accuracy. The Chinese SQVD conformed to a unidimensional structure with excellent reliability and validity. Person and item reliabilities were 0.85 and 0.99, respectively, indicating, high stability. Person and item separation indices were 2.37 and 11.54, respectively, signifying strong differentiation ability. Retest reliability was 0.917, further emphasizing the stability of the scale. The area under the receiver operating characteristic curve was 0.908 (95% CI: 0.854, 0.962), with a cutoff value of 7.5 and Youden index of 0.733, highlighting the scale’s high diagnostic accuracy. The translated and culturally adapted Chinese SQVD demonstrated excellent psychometric properties. With streamlined items, short assessment time, and high efficiency, the scale is a stable and reliable clinical tool for detecting a variety of conditions related to refractive, regulatory, and binocular vision dysfunction.

## 1. Introduction

Visual dysfunctions (VDs) encompass varying degrees of visual function impairment, leading to compromised visual fields, diminished visual acuity, and inability to achieve normal levels of visual function, ultimately impacting daily life.^[1]^ Research indicates that VDs have an array of negative impacts on patients’ quality of life, such as limitations in performing daily activities, educational and learning constraints, and reduced participation in social engagements.^[2]^ Many VD problems can be addressed through refractive correction or functional exercises, while certain cases may necessitate medication or surgical intervention. Unfortunately, in some instances, full restoration of vision may not be achievable even with these treatment measures.^[1]^

The World Health Organization’s August 2014 update on Visual Impairment and Blindness (World Health Organization Fact Sheet No. 282) states that >80% of global VD cases are preventable or treatable.^[3]^ Despite this, millions remain at risk of vision loss due to a lack of access to eye care services.^[4]^ Early assessment of a patient’s extend of VD is therefore of crucial importance in clinical practice. To address this need, Professor Mario Cantó and his team in Spain developed the symptom questionnaire for visual dysfunctions (SQVD) scale to assess the subjective quality of vision in visually impaired patients, going on to comprehensively analyze its psychometric properties including accuracy, validity, and reliability.^[5]^ The SQVD scale demonstrated the ability to detect visual symptoms associated with various refractive errors, as well as accommodative and binocular dysfunctions, exhibiting higher sensitivity and reliability compared to traditional vision tests. Based on a Rasch model, the scale yields statistical data regarding the presence and frequency of visual symptoms associated with visual abnormalities, containing important information about the degree of individual VD. These qualities make SQVD a valuable diagnostic tool for clinicians.^[6]^

The aim of this study was to translate, sinicize, and cross-culturally adapt the original Spanish SQVD. The Rasch model was employed to analyze the adapted version, ensuring that the resulting tool possesses reliable, valid measures for detecting visual symptoms associated with refractive, regulatory, and binocular dysfunctions in the Chinese context.

## 2. Methodology

### 2.1. Instruments

Two main assessment instruments were utilized in this study. Firstly, the SQVD, which consists of 14 items that facilitate a comprehensive examination over a broad range of symptoms associated with VD. The finalized 14 items in the SQVD encompass aspects like blurred vision, binocular vision problems, ocular irritation, concentration difficulties, reading problems, and headaches. To gauge the frequency of each symptom, the scale is structured with 3 levels: (1) “No,” indicating that the symptom never occurs, scored as 0; (2) “Occasionally or often,” signifying that the symptom occurs occasionally (at least once every 15 days) or once to twice per week, with a score of 1; (3) “Almost always,” indicating that symptoms occur nearly every day, scored as 2. As the score for each entry ranges from 0 to 2, the total SQVD score for these 14 items ranges from 0 to 28.

The second primary tool is the General Information Questionnaire, jointly compiled by members of the research team after an extensive literature review. This questionnaire covers topics such as patients’ age, gender, education level, presbyopia status, and binocular visual function test results.

### 2.2. Stages of research

This study was conducted into 2 stages. In the first stage, qualitative research methods were employed for precise translation of the SQVD scale, ensuring its suitability to the target cultural context. This involved aligning the scale’s content and presentation with the cultural concepts and cognitive styles of the intended audience. In the second stage, we explored the psychometric properties of the Chinese version of the SQVD scale in depth based on extensive data collection and analysis, aiming to assess its validity and reliability.

#### 2.2.1. Stage 1: Chinese translation and cross-cultural adaptation

##### 2.2.1.1. Scale translation

To verify the accuracy and consistency of the original scale, we first contacted Professor Mario Cantó by email and obtained official authorization from his team. Next, we translated and back-translated the scale with reference to the Modified Brislin Translation Model.^[7]^ Two bilingual individuals, “Positive Translator 1” and “Positive Translator 2,” translated the source scale E0 into Chinese versions C1 and C2. After this initial translation, we conducted an intragroup discussion and integrated the perspectives of both translators to obtain C3.

Following this, “Back-translator 3,” a Master’s student in English with overseas study experience, back-translated C3 to create the back-translated version E1. “Monolingual Translator 4” then compared the source scale and back-translated scale. When differences were found, Monolingual Translator 4 discussed them with the 3 bilingual translators to facilitate a shared understanding of the correct meaning. Version E2 was secured after repeated discussion until reaching a consensus among the 3 bilingual translators and Monolingual Translator 4. Lastly, the 3 bilingual translators collaborated with Shandong Wanteng Translation Service Co., Ltd. to discuss the translation of E2 into the final Chinese version C4.

Through this series of rigorous processes, we ensured the accuracy and consistency of the scale as a reliable basis for subsequent research and analysis.

##### 2.2.1.2. Cultural adjustment

To ensure the precision and applicability of the Chinese version of the SQVD, C4, a cultural adjustment process was conducted. This involved experts and doctors in the fields of optometry and eye care, with a working experience of between 12 and 28 years and an average of (15.77 ± 7.498) years, all holding Associate Professor or higher-level titles and a minimum of a Bachelor’s degree. Throughout this adjustment process, we comprehensively evaluated and modified each item of C4 with respect to the following 4 key aspects.^[8]^

① Is the content of the item clear, accurate, and easy to understand?

② Does the content fit the theme and meet the assessment requirements?

③ Is the content consistent with the original English version?

④ Are there any items that need to be added, deleted, modified, or merged?

Through a series of assessments, we successfully optimized the Chinese SQVD to align it with the cultural background and practical application needs. Considering the experts’ insights, we modified some entries to resonate with the daily language habits of the Chinese populace and their understanding of VDs. For instance, Item 9’s phrasing “have neck or back pain” was revised to “with neck or back pain” in accordance with the experts’ recommendations.

##### 2.2.1.3. Pre-survey of target population

Prior to the formal survey, in July 2023, a convenience sampling method was adopted to select 30 patients from the ophthalmology outpatient clinic of a tertiary hospital in Hohhot City for a pre-survey questionnaire. Following the questionnaire’s completion, a brief inquiry was conducted to gauge the participants’ comprehension of the scale’s indicators, the content of each item, and each option, with the findings duly recorded. We then refined the scale based on the patients’ feedback and an additional expert consultation to derive the final Chinese version of the SQVD C5. Within 2 weeks of questionnaire completion, patients were invited to revisit the same questions to evaluate the retest reliability of the scale.

Modifications were made based on patient feedback, for example, to Item 5: “Reread the test” was adjusted to “reading multiple times” as per the comments received.

#### 2.2.2. Stage 2: assessment of Chinese SQVD’s psychometric properties

##### 2.2.2.1. Study population and data collection methodology

A convenience sampling method was used to select patients who visited the ophthalmology clinic of a tertiary-level hospital in Hohhot City from August to December 2023 for participation in this study. Inclusion criteria encompassed individuals: (1) aged > 14 years old; (2) who underwent ophthalmological examination of binocular visual function (refractive, convergence-divergence, and accommodation functions); (3) voluntarily participating in the study by signing an informed consent form. Exclusion criteria involved: (1) history of refractive surgery or taking vision-altering drugs; (2) patients with dry eye diseases; (3) the presence of mental disorders, hearing or communication disorders; (4) those with organic ocular lesions.

The study was approved by the Ethics Committee of the Affiliated Hospital of Inner Mongolia Medical University and aligned with the principles of the Declaration of Helsinki. Before commencing the survey, we first obtained the patients’ contact information and explained to them in detail its purpose and significance. Before distributing the questionnaire, we reiterated its purpose and the meaning of each item to ensure the participants’ full understanding. For patients under 18 years of age, guardians were required to sign the informed consent form alongside them.

The Rasch model has no clear formula or criteria; decisions are generally based on the nature of the research question, the availability of data, and the complexity of the model.^[9]^ Larger sample sizes generally lead to more effective model estimations. Lerdal^[10]^ suggests a minimum sample size of at least 150 to obtain a stable model within the ±0.5 logit interval. Accounting for a 10% sample attrition rate, the sample needs to be at least 165 cases. A total of 270 questionnaires were distributed in this study and 252 valid questionnaires were collected, yielding a valid recovery rate of 93.33%.

### 2.3. Statistical methods

The data obtained were entered and double-checked in Excel. Statistical analysis and data processing were conducted in SPSS 24.0 and Winsteps 3.74.0. General information was recorded in terms of the number of cases and composition ratio.

Rasch analysis was performed using Winsteps software. The partial credit model was used to analyze the SQVD scale for response category functionality, fit statistics, unidimensionality, person and item reliability, separation, targeting, and differential item functioning (DIF).

*Response category functionality*: Rating scale analysis was employed to assess the rationality of the scoring options for the three-point scale. Criteria included (1) options frequency exceeding 10, a monotonically increasing mean difficulty value, outfit NMSQ below 2, monotonically increasing rank difficulty, and a difference in difficulty between adjacent ranks on the three-point scale exceeding 1.4 logits.^[11]^

*Fit statistics*: Infit MNSQ and Outfit MNSQ were used to assess the fit of the test items to the subjects’ ability, considering the abnormal response of test items to said ability. Desired values for Infit MNSQ and Outfit MNSQ close to 1 indicate a well-fitted model, where less-challenging entries are less likely to be answered incorrectly, and vice versa. Values below 0.50 indicate possible redundancy, while values above 1.50 may indicate that and item measures something inconsistent with the overall content of the scale. Thus, the fit statistic should fall within the range of 0.50 to 1.50.^[12]^ The optimal range for a standardized fit is between ‐2 and 2.

*Unidimensionality*: This instrument measures only one underlying trait or ability, with variance in the data entirely explained by this singular dimension.^[13]^ When employing principal component analysis with scale test data, the first principal component (i.e., the dominant dimension) should explain >50% of the original variance, and the residual eigenvalues of the first component should range from 1.4 to 2.1.^[14]^

*Reliability*: To evaluate the reliability of a measurement tool, People Reliability was used to assess the stability and consistency exhibited by subjects in completing the test^[15]^ and Item Reliability was used to assess the reliability of the measurement tool.^[15]^ Values for both metrics range from 0 to 1, with reliability exceeding 0.70 generally considered indicative of good reliability.^[16]^

*Separation*: Discriminability was assessed using the Person Separation Index (PSI). A larger PSI value indicates better discrimination between individuals.^[17]^ When the PSI value is >2, the instrument discriminates between individuals at different levels effectively. Structural validity was assessed using the Item Separation Index, which indicates better differentiation between items when it has a larger value.^[18]^ The PSI is the most important indicator of the structural validity of the instrument.

*Targeting*: Utilizing the person-item map involves aligning individual ability and entry difficulty on the same scale. By analyzing the difference in logits between the measured values of all items based on their positions on the scale, this instrument provides a clear depiction of the distribution of subjects’ ability values and entry difficulty values. Subsequently, it allows an assessment of whether the difficulty settings satisfy the needs of subjects with different abilities.^[19]^ If neighboring items have more than a 0.5 logit difference, then there is a potential need for additional items to fill the gap. Conversely, items with the same difficulty may be redundant.^[20]^

*Differential item functioning*: This instrument assesses whether differences exist in the test item’s measurement properties of the subject’s ability between different groups of subjects.^[21]^ DIF reveals potential unfairness or bias in a test item across different groups. A contrast is deemed substantially different if the absolute value of the difference between the 2 groups is >1 logit, indicating bias in the item.^[22]^

The data were analyzed for internal consistency, correlation, and retest reliability using SPSS 24.0 software. The results of binocular visual function tests were used as the gold standard for plotting subjects’ work characteristic curves and evaluating the overall accuracy of the test.

*Internal consistency*: McDonald Omega coefficient was employed to measure the internal consistency of the scale. A larger McDonald Omega value indicates better homogeneity of the scale, and signifies reliability when it is at least 0.7.^[23]^

*Retest reliability*: Assuming a stable test, the same test taken by the same subjects at different time points should yield highly similar results. To quantify this similarity, we calculated the Pearson correlation coefficient between the initial test and retest. A correlation coefficient close to 1 indicates high consistency, thus demonstrating high retest reliability.^[24]^

*Correlation analysis*: Spearman correlation coefficients were calculated between the scores of each entry and the total score. These coefficients, commonly used to measure the degree of linear correlation between 2 variables in the same set of variables, assist in determining the consistency and correlation of each item with the questionnaire as a whole. A correlation coefficient of >0.3 indicates a high degree of consistency and correlation with the questionnaire.^[25]^

*Receiver operating characteristic (ROC) curve*: The subjects’ binocular visual function examination results were considered as the baseline, and categorized into symptomatic and asymptomatic groups based on the presence or absence of symptoms. ROC analysis was then performed using the raw SQVD scores of the patients in both groups. The analysis yielded the Area Under the Curve of the ROC curve, with higher Area Under the Curve (closer to 1) indicating better accuracy of the SQVD in detecting symptoms.^[26]^ The cutoff point was identified considering a balance between specificity (S) and sensitivity (SP). The Youden index, which aims to maximize the difference between S and SP, was employed to determine the optimal cutoff value; it was calculated as Youden index = Sensitivity + Specificity ‐ 1. Analyzing the S and SP of each point on the ROC curve revealed the cutoff value that maximizes the Youden index for optimal diagnostic outcomes.^[27]^

## 3. Results

### 3.1. Sample characteristics

Table 1 outlines the demographic characteristics of the study population. Among the 252 subjects, 142 are male and 110 are female. At the time of their participation, their mean age was 37.44 ± 13.98 years, 30.1% had normal visual function in both eyes, 51.6% suffered from refractive dysfunction, 8.7% suffered from accommodation dysfunction, 7.2% suffered from convergence-divergence dysfunction, and 2.4% suffered from accommodation + convergence-divergence anomalies. Additionally, 18.9% of the subjects suffered from presbyopia.

**Table 1:** Sociodemographic and clinical characteristics of participants (n = 252).

Classification	Number (%) of cases
*Gender*	
Male	142 (56.3)
Female	110 (43.7)
*Education level*	
Primary and below	58 (23.0)
Junior	70 (27.8)
High school	73 (29.0)
University and above	51 (20.2)
*Marital status*	
Married	149 (9.1)
Divorced	5 (2.0)
Widowed	4 (1.6)
Never married	94 (37.3)
*Payment method*	
Self-paying	56 (22.2)
Medical insurance	196 (77.8)
*Monthly income*	
＜2000¥	49 (19.4)
2000-4999¥	79 (31.3)
5000-7999¥	91 (36.2)
＞8000¥	33 (13.1)
*Binocular visual function test*	
Normal	76 (30.1)
Refractive dysfunction	130 (51.6)
Regulation dysfunction	22 (8.7)
Convergence and divergence dysfunction	18 (7.2)
Adjustment + abnormal convergence and divergence	6 (2.4)
*Presbyopia*	
Yes	40 (18.9)
No	212 (81.1)

### 3.2. Functionality of the response categories

**Figure 1.**
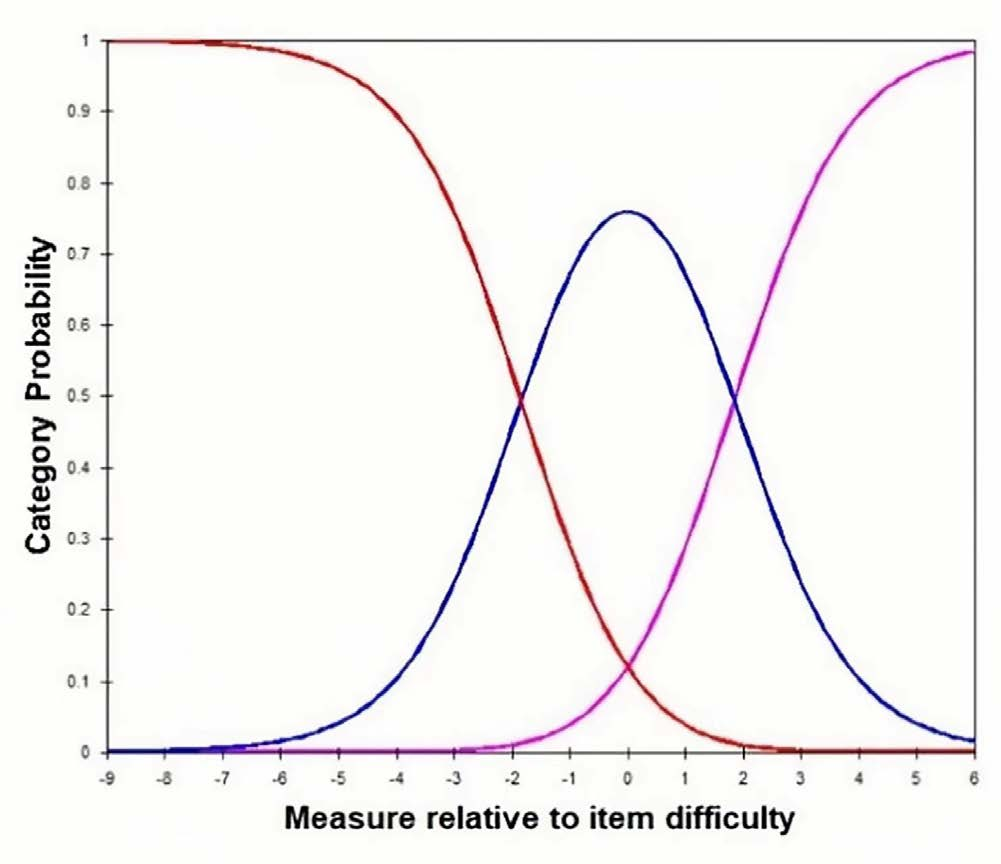
Option probability curve.

As shown in Table 2, the frequencies of the response categories were all >10. Both the mean difficulty value and the ranked difficulty value increased monotonically, indicating that the options were measured with a single choice of assessment scale and were consistent with the latent variables being measured. The Outfit MNSQs for the options were all less than the criterion value of 2, signifying that the data meet the assessment requirements of the Rasch model. Combined with the option probability curves in Figure 1, the questionnaires demonstrate good measurement precision for subjects with intermediate levels of ability, with low measurement error for the sample size of this study.

**Table 2:** Category ratings on three-point scale.

Options	Frequency	Average difficulty	Outfit MNSQ	Degree of difficulty
0	962	‐2.64	0.98	None
1	1803	0.06	0.99	‐1.84
2	763	1.84	1.00	1.84

### 3.3. Unidimensionality

The raw variance explained by the first component was 52.5%, and the eigenvalue of the standardized residuals of the first component was 1.8 (Table 2), which are within the desirable range, indicating that the Chinese version of the SQVD scale complied with the unidimensionality requirement.

### 3.4. Reliability and separation

The People Reliability (0.85) and Item Reliability (0.99) of the SQVD scale (Table 3) indicate that the scale has good reliability; the PSI (2.37) indicates that the Chinese version of the SQVD can be classified into at least 3 different levels of competence in the subject population. The Item Separation Index (11.54) indicates that the Chinese version of the SQVD possesses at least 11 levels of difficulty, further indicating that the results obtained from the scale are reliable.

**Table 3:** Fit parameters of Rasch model.

Parameter	Reference value	SQVD scale
*Reliability*		
Person reliability (PR)	>0.7	0.85
Item reliability (IR)	>0.7	0.99
*Separation*		
Person Separation Index (PSI)	>2	2.37
Item Separation Index (ISI)	>3	11.54
*Person fit*		
Infit MNSQ	>0.5 and <1.5	0.99
Outfit MNSQ	>0.5 and <1.5	1.02
*Item fit*		
Infit MNSQ	>0.5 and <1.5	1.00
Outfit MNSQ	>0.5 and <1.5	1.02
*Target* (*mean difference between people and items*)	<1	‐0.29
*Principal component analysis* (*PCA*)		
Raw variance explained by measures	>50%	52.5
Unexplained Variance in 1st contrast	>1.4 and <2.1	1.8
Differential item functioning (DIF)	<0.5, *T* < 2	No

### 3.5. Fit statistics

The majority of the values for each parameter among both fit situation indicators, Infit and Outfit, are within the ideal range of 0.5 to 1.5. This suggests that the data for this measurement align well with the model, indicating an accurate estimation of the subjects’ ability levels and the difficulty of the items (Table 4).

**Table 4:** Chinese SQVD scale fitted to Rasch model.

Item	Infit		Outfit		Person measure
	MNSQ	ZSTD	MNSQ	ZSTD	
Item 1	1.05	0.6	1.14	0.7	0.36
Item 2	1.13	1.7	1.12	1.6	0.59
Item 3	0.70	‐3.6	0.67	‐3.6	0.64
Item 4	0.91	‐1.2	0.89	‐1.3	0.73
Item 5	0.97	‐0.3	0.96	‐0.5	0.62
Item 6	0.92	‐1.0	1.03	0.2	0.43
Item 7	1.10	1.0	1.05	0.5	0.62
Item 8	1.29	2.6	1.47	2.7	0.51
Item 9	0.90	‐1.3	0.90	‐1.2	0.65
Item 10	0.95	‐0.7	0.95	‐0.6	0.55
Item 11	1.00	0.1	1.00	0.0	0.62
Item 12	1.09	1.2	1.09	1.1	0.60
Item 13	0.97	-0.4	0.96	‐0.5	0.67
Item 14	1.01	0.2	1.02	0.3	0.53
Mean	1.00	-0.1	1.02	‐0.1	–
SD	0.13	1.5	0.17	1.4	–

### 3.6. Differential item functioning

In examining DIF on the Chinese version of the SQVD scale, both gender (male and female) and presbyopia (presence and absence) were considered. After rigorous analyses, no significant DIF was identified, indicating that the scale has strong measurement equivalence across these 2 categories.

### 3.7. Targeting

**Figure 2.**
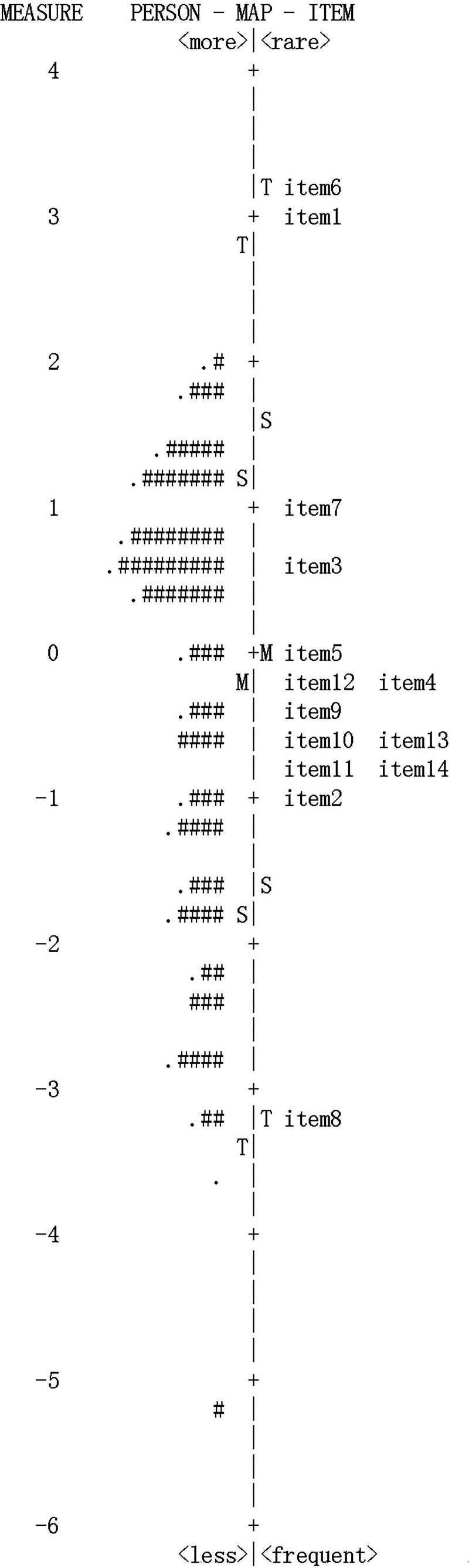
Person-item map of SQVD. SQVD = symptom questionnaire for visual dysfunctions.

*Note*: The number of subjects is marked on the left side of the dotted line, with “#” denoting 2 persons and “‐” denoting one person. “M” indicates the mean, with human ability on the left side and item difficulty on the right. “S” represents 1 standard deviation from the mean, and “T” represents 2 standard deviations; items closer to the top indicate more frequently experienced symptoms, and items of greater difficulty.

The mean score for difficulty was set to 0 in the Rasch model. The target value of ‐0.29, as shown in Figure 2, suggests that a large number of patients were able to obtain better measures. However, Item 6 (3.21) and Item 1 (3.28) reveal that even patients with more severe symptoms do not readily exhibit them. Conversely, the symptoms associated with Item 8 (‐3.28) were possible in both mildly and severely symptomatic patients. This suggests that these 3 entries have limitations in differentiating between patients with varying levels of symptoms.

### 3.8. Classical test theory assessments

*Internal consistency*: The McDonald Omega for the Chinese version of the SQVD was 0.853, indicating that the scale has good reliability.

*Retest reliability*: A follow-up assessment was distributed via telephone after 2 weeks, involving 30 patients, indicating a retest reliability of 0.917. The data conformed to normality as per the Pearson correlation coefficient, indicating strong stability for the scale.

*Correlation analysis*: The correlation coefficients between each entry of the Chinese version of the SQVD and the total score ranged from 0.374 to 0.704 (Table 5) Spearman correlation coefficient was used in this case, as the data did not exhibit normality. The items meet relevant criteria and were significantly correlated with the total score.

**Table 5:** Correlations of Chinese SQVD with total score (*r*).

	1	2	3	4	5	6	7
SQVD total score	0.374**	0.592**	0.606**	0.704**	0.611**	0.488**	0.650**
	8	9	10	11	12	13	14
SQVD total score	0.524**	0.627**	0.516**	0.587**	0.617**	0.648**	0.500**

** Indicates *P* ﹤ .01.

**Figure 3.**
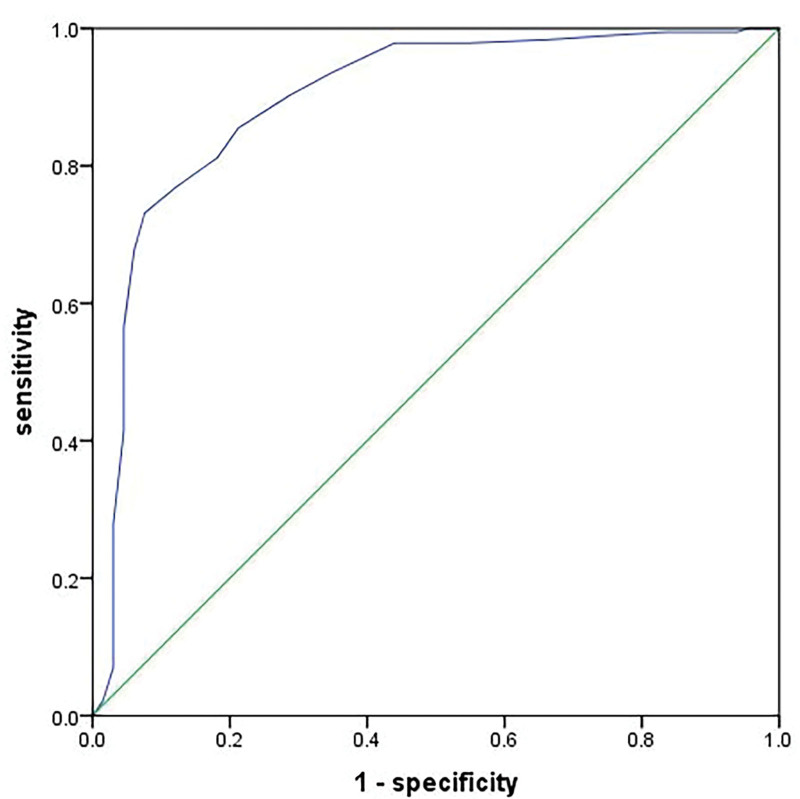
ROC curve for SQVD questionnaire. ROC = receiver operating characteristic, SQVD = symptom questionnaire for visual dysfunctions.

*ROC curve*: As per the ROC curve analysis (Fig. 3), the sensitivity of the Chinese version of the SQVD in predicting symptom onset was 90.8% (95% CI: 0.854, 0.962). The optimal cutoff value was 7/8, corresponding to a Jordon index of 0.733, with diagnostic significance scores of 7 or 8.

## 4. Discussion

Based on the Brislin translation model, the Spanish version of the SQVD scale was translated, back-translated, and cross-culturally debugged. Combining the outcomes of a pre-survey and expert consultations, we made appropriate modifications to individual items to make align them with the Chinese cultural context. After rigorous Rasch analysis, we confirmed that the Chinese version of the SQVD scale has excellent an person reliability score, item reliability score, person separation index, and item separation index. These findings underscore the scale’s high reliability and robust psychometric properties. The data aligned with the Rasch model requirements, with all fitted data falling within expected interval ranges. While some items posed challenges in terms of difficulty levels, they comprehensively covered patients’ symptoms and effectively detected and differentiated them. Considering the scale’s fit, reliability, validity, and other factors, we decided to retain the original 14 items to ensure stability and consistency.

The Chinese version of SQVD also demonstrated good internal consistency and retest reliability. These qualities allow for multiple assessments of the same patient to accurately monitor treatment effects. Additionally, the scale exhibited significant diagnostic accuracy in identifying various types of VD-related symptoms. ROC analysis revealed that scores at or above 7 or 8 effectively correlate with subjects’ visual symptoms and specific VDs.

In summary, the Chinese version of the SQVD exhibits strong psychometric properties with its 14 items, ensuring a quick and effective assessment of patients with VDs. It may serve as a reliable and valid tool for measuring visual symptoms. A previous study utilizing this scale adopted the Delphi method^[28]^ obtaining a more comprehensive diagnostic perspective regarding the symptoms of VD patients. The results of this study not only enhance the reliability and validity of the Chinese version of the SQVD but also broadens its applicability and clinical significance.

In practice, the Chinese version of the SQVD scale can assist healthcare professionals in quickly assessing a patient’s degree of VD. This, in turn, aids in the formulation of more tailored rehabilitation interventions. For patients, the scale’s results offer personalized treatment recommendations, facilitating the recovery of their visual function. Furthermore, the scale can be utilized to monitor the effectiveness of treatments, providing an objective basis for assessment by both doctors and patients.^[6]^

Future research endeavors should focus on further validating the applicability and cross-cultural consistency of the Chinese version of the SQVD scale. As the scale was developed and debugged within the Chinese context, more research is yet warranted to demonstrate its suitability in other cultural settings. Future studies can also explore the correlations between the Chinese version of the SQVD scale and other visual function assessment tools^[29,30]^ The scale’s applicability across different age groups, genders, and education levels should also be investigated to enhance its universality and relevance, thereby improving its comprehensive utility in clinical practice. For special groups of patients with VD (e.g., children, the elderly), the scale may need to be customized and adapted according to their unique cognitive and perceptual abilities. We hope that the results of this study will provide valuable references for experts, scholars in related fields, and clinical healthcare professionals, contributing collectively to improving the quality of life for patients with VDs.

## 5. Summary

In this study, the reliability and validity of a novel Chinese version of the SQVD scale were systematically examined after normatively sinocizing the original scale and incorporating item response theory and classical measurement theory. The Chinese SQVD contains 14 items, exhibiting a unidimensional structure with high reliability and validity as well as favorable person separation index performance. Importantly, the absence of local dependence among items enhances the scale’s effectiveness in assessing symptoms in patients with VDs.

The Chinese SQVD demonstrated exceptional accuracy in diagnosing various types of VD-related symptoms. Rasch model analysis further confirmed the scale’s reasonable ordering, moderate overall difficulty, and appropriate difficulty gaps between adjacent options.

In future studies, exploring the synergistic effects of the Chinese version of SQVD with other visual function scales and ophthalmology-related quality-of-life scales can be pursued to advance the development of visual impairment assessment tools, ultimately enhancing the quality of life for patients with visual impairment.

## Author contributions

**Conceptualization:** Xianrong Yang, Zizhong Zhang, Weiwei Jiang.

**Data curation:** Yufeng Wang.

**Formal analysis:** Yufeng Wang.

**Funding acquisition:** Xianrong Yang.

**Investigation:** Yufeng Wang.

**Methodology:** Xianrong Yang, Xin Jia, Xianrong Yang.

**Project administration:** Xianrong Yang, Hongai Liu.

**Resources:** Xianrong Yang.

**Supervision:** Xianrong Yang, Xin Jia, Xianrong Yang.

**Writing – original draft:** Yufeng Wang, Zizhong Zhang, Weiwei Jiang.

**Writing – review & editing:** Xianrong Yang, Zizhong Zhang, Weiwei Jiang.
